# Multifunctional Nanoplatforms Bridging Diagnostics and Therapeutics in Cancer

**DOI:** 10.3390/mi16121323

**Published:** 2025-11-26

**Authors:** Hossein Omidian, Erma J. Gill

**Affiliations:** Barry and Judy Silverman College of Pharmacy, Nova Southeastern University, Fort Lauderdale, FL 33328, USA; eg1262@mynsu.nova.edu

**Keywords:** synergistic tumor imaging, nanotheranostics, multimodal diagnostics, tumor microenvironment, translational oncology

## Abstract

Accurate tumor visualization remains a central challenge in oncology, as single-modality imaging often lacks the depth, sensitivity, and specificity needed for precise therapeutic guidance. Nano-theranostic platforms address this by combining multimodal imaging with tumor-responsive activation and therapeutic functions within a single system. Advances in carbon-based nanomaterials, metallic and metal oxide nanoplatforms, polymeric and lipid carriers, and biomimetic architectures enable integration of fluorescence (FL), near-infrared II fluorescence (NIR-II FL), photoacoustic (PA), magnetic resonance (MRI), computed tomography (CT), and ultrasound (US) imaging for comprehensive anatomical, functional, and molecular tumor characterization. Coupled with photothermal therapy (PTT), photodynamic therapy (PDT), chemo-dynamic therapy (CDT), ferroptosis induction, metabolic modulation, gas-based therapeutics, and immune activation, these nanoplatforms transform imaging from a passive diagnostic tool into an active, feedback-regulated therapeutic modality. This review outlines the mechanistic foundations, integrated functionalities, and preclinical significance of synergistic imaging-guided nano-theranostics. We also highlight emerging priorities—including adaptive closed-loop platforms, streamlined multifunctional designs, immunotherapy integration, and scalable, biocompatible manufacturing—to advance clinically viable nano-theranostics for precision oncology.

## 1. Introduction

Cancer remains one of the leading causes of morbidity and mortality worldwide, with therapeutic outcomes frequently limited by resistance to conventional modalities such as chemotherapy and radiotherapy [1,2,3,4,5,6]. Although widely used in clinical practice, these treatments are hindered by systemic toxicity, insufficient tumor selectivity, and poor performance against resistant or recurrent malignancies. At the same time, diagnostic imaging—despite its essential role in tumor detection and staging—often lacks the sensitivity and spatial resolution needed for precise treatment guidance. These challenges underscore the pressing need for integrated platforms capable of unifying diagnosis and therapy within a single system.

Nano-theranostics have emerged as such a platform, offering the ability to combine diagnostic and therapeutic functions at the nanoscale to support real-time, image-guided precision oncology [7,8,9,10,11,12,13]. By leveraging diverse classes of nanomaterials—including carbon-based, metallic, polymeric, biomimetic, and supramolecular constructs [5,14,15,16,17,18,19,20,21,22,23,24,25,26,27,28,29,30,31,32,33,34,35,36,37,38]—these multifunctional platforms integrate multimodal imaging (fluorescence, photoacoustic, magnetic resonance, computed tomography, and ultrasound) with tumor-responsive activation and site-specific drug delivery. Features such as hypoxia, acidosis, and redox imbalance enable microenvironment-selective activation, improving therapeutic specificity while reducing systemic toxicity [35,39,40,41,42].

Quantum dot (QD)–based nanomaterials represent a particularly promising frontier. Their high optical stability and tunable emission properties support sensitive biomarker detection and targeted therapeutic applications. In prostate cancer, QD-enabled biosensing platforms exhibit strong specificity and compatibility with biological fluids, while advances in green synthesis and smartphone-integrated devices facilitate decentralized, real-time diagnostics [43]. In breast cancer, QDs improve both early detection and drug delivery, particularly for aggressive subtypes such as triple-negative breast cancer (TNBC), although challenges related to biocompatibility and translational scalability persist [44].

Beyond diagnostics, the integration of multimodal imaging with therapeutic activation has positioned nanotheranostics as active agents of therapy. Hybrid systems combining PTT and PDT modalities achieve synergistic tumor ablation while enabling real-time monitoring and spatiotemporal control [45]. When further coupled with chemotherapy, gene silencing, or radio sensitization, these platforms enhance therapeutic efficacy, and immune-responsive nanostructures offer additional opportunities for durable tumor suppression and systemic antitumor immunity [46].

This review provides a comprehensive synthesis of these advances, structured around five major themes: (i) clinical motivations and biological barriers; (ii) design strategies for multifunctional nanoplatforms; (iii) mechanistic functional pathways; (iv) progress in multimodal imaging; and (v) synergistic therapeutic modalities. Alongside key preclinical findings and translational milestones, we critically examine current limitations and outline emerging directions for developing adaptive, safe, and clinically actionable nanotheranostics systems. Through this integrated framework, we aim to offer a strategic roadmap for advancing nanotheranostics from mechanistic innovation toward clinical implementation in precision oncology.

## 2. Current Barriers in Cancer Therapy

### 2.1. Limitations of Conventional Therapies

Chemotherapy and radiotherapy remain central pillars of oncologic treatment, yet both face persistent limitations, including systemic toxicity, suboptimal tumor accumulation, and inadequate tumor selectivity. These challenges contribute to therapeutic failure, disease recurrence, and poor overall survival, particularly in therapy-resistant malignancies such as triple-negative breast cancer and hepatocellular carcinoma [1,2,3,4,5,6]. The nonspecific cytotoxicity of these modalities also compromises healthy tissue integrity, further narrowing their therapeutic window.

### 2.2. Tumor Microenvironment-Driven Barriers

Multiple biological and physical features of the tumor microenvironment (TME) impede treatment efficacy. Hypoxia—a hallmark of many solid tumors—reduces the effectiveness of oxygen-dependent therapies, including radiotherapy and PDT [36,47,48]. Dense desmoplastic stroma, as seen in pancreatic ductal adenocarcinoma, restricts vascular perfusion and limits intratumoral drug distribution [49]. Elevated intracellular glutathione levels and the immunosuppressive activity of tumor-associated macrophages further promote redox homeostasis and immune evasion, both of which are closely tied to resistance against conventional and targeted therapies [50,51,52,53].

### 2.3. Challenges in Phototherapies and Combination Approaches

While PTT and PDT offer minimally invasive treatment options, both are constrained by technical and biological limitations. PDT efficacy depends on sufficient oxygen availability within the TME, while PTT may generate uneven heat distribution, increasing the risk of incomplete ablation and thermal injury to surrounding tissues [19,26,54,55,56]. Combination strategies such as chemo-PTT and chemo-PDT seek to enhance therapeutic synergy, yet their effectiveness is often diminished by premature drug release, inadequate tumor retention, and limited intratumoral cooperation among therapeutic components [31,40,57]. These shortcomings highlight the need for platforms capable of coordinated delivery, controlled activation, and spatially precise therapeutic action.

### 2.4. Diagnostic and Imaging Limitations

Current imaging modalities frequently lack the sensitivity, spatial resolution, and tissue penetration depth required for accurate tumor diagnosis and intraoperative guidance. Fluorescence imaging is hindered by high background autofluorescence and shallow tissue penetration, reducing contrast and impairing tumor delineation [58]. MRI, despite its clinical utility, often provides modest contrast enhancement and limited molecular specificity [59]. Such limitations compromise real-time surgical navigation and post-treatment monitoring, increasing the likelihood of residual disease, incomplete ablation, and disease progression [25,29,60,61,62,63].

### 2.5. Barriers to Translational Nanomedicine

Despite significant advances, many nanotheranostics systems encounter obstacles that impede clinical translation. Structural complexity, incomplete biodegradability, and dependence on the enhanced permeability and retention (EPR) effect contribute to variable tumor accumulation and off-target distribution [7,8,9,10]. Even sophisticated stimuli-responsive platforms may demonstrate limited activation within the heterogeneous and dynamic TME, reducing their ability to coordinate diagnostic and therapeutic functions [11,12,13]. Additionally, regulatory, manufacturing, and scalability challenges further complicate the transition from preclinical research to clinical application.

Collectively, these barriers—spanning therapeutic toxicity, biological resistance, diagnostic insufficiency, and translational constraints—underscore the need for next-generation nanotheranostics platforms engineered for enhanced tumor specificity, integrated functionality, and clinical viability.

## 3. Design Strategies for Multifunctional Nanoplatforms

### 3.1. Carbon-Based Nanostructures

Carbon nanomaterials offer exceptional structural and functional versatility due to their tunable surface chemistry, π-conjugated frameworks, and diverse architectures. Heteroatom-doped carbon dots (S,N-CDs), for example, exhibit enhanced photo reactivity and improved optical characteristics through precise atomic-level doping [14]. Green-synthesized polyphenolic green-emitting carbon quantum dots (g-CQDs) demonstrate pH-responsive, electrostatically driven drug-loading capabilities that support controlled delivery [20]. Hollow mesoporous carbon spheres (HMCS) provide a hydrophobic interior suitable for drug encapsulation and facilitate efficient, low-temperature photothermal therapy [26]. Tubular systems such as multi-walled carbon nanotubes further illustrate the loading potential of carbon scaffolds; when functionalized with hyaluronic acid and π–π stacked drug molecules, they achieve high payload capacity and improved targeting [27]. Collectively, these platforms highlight how heteroatom doping, controlled porosity, and π–π interactions contribute to enhanced drug delivery, targeting specificity, and therapeutic responsiveness.

### 3.2. Metal and Metal Oxide Platforms

Metal- and metal oxide–-based nanoplatforms employ core–shell architectures, defect engineering, and compositional tuning to integrate diagnostic and therapeutic functions. Platinum (*Pt*)-tipped gold (*Au*) nanorods encapsulated within Zeolitic Imidazolate Framework-8 (ZIF-8) are notable examples, combining plasmonic photothermal effects with catalytic hypoxia modulation and chemotherapeutic co-delivery [17]. Defect-rich iron (*Fe*)-doped molybdenum oxide (*MoO_x_*) nanowires demonstrate redox-driven therapeutic activity and pH-responsive biodegradation [18], whereas gadolinium (*Gd*)-doped iron(II,III) oxide (*Fe_3_O_4_*) nanoparticles enable dual-mode magnetic imaging coupled with photothermal-enhanced chemotherapy [64]. Redox-sensitive manganese dioxide (*MnO_2_*) shells offer glutathione-triggered disintegration and membrane cloaking, supporting immune evasion and localized drug release [21]. These examples illustrate how alloying, heteroatom doping, and surface-shell engineering expand the functional capabilities of metal-based nanostructures for integrated cancer theranostics.

### 3.3. MOFs and Coordination Assemblies

Metal–organic frameworks (MOFs) and related coordination assemblies provide porous, crystalline scaffolds capable of high drug loading and modular functionalization. Hybrid platforms such as iron- and copper-doped MOF-199 modified with polydopamine (*Fe/Cu-MOF-199@PDA*) incorporate catalytic metals and enzymes within a stabilized polymer matrix, enabling redox modulation and multimodal therapeutic responses [32]. Bimetallic UiO-67 derivatives leverage dual-metal coordination to enhance drug encapsulation and enable efficient presentation of targeting ligands [24]. Post-synthetic transformations—such as converting ZIF-67 into cobalt(II,III) sulfide (*Co_3_S_4_*) nanozymes—illustrate how targeted doping can generate catalytically active theranostic systems [33]. In other designs, drugs such as doxorubicin (DOX) are directly coordinated within the MOF architecture, as demonstrated by *Fe^3+^*–DOX–lipoic acid polymeric networks responsive to intracellular redox gradients [5]. These constructs underscore the power of coordination chemistry in enabling enzyme-mimetic catalysis, redox-activated therapy, and stabilized drug integration within multifunctional nanoplatforms.

### 3.4. Silica and Organosilica Systems

Silica-based platforms—including mesoporous and organo-silica derivatives—provide robust, structurally adaptable matrices for integrating multiple functional components. The upconversion nano-jelly hydrogel (UCNJ) tri-layer system exemplifies this hierarchical design: an upconversion nanoparticle (UCNP) core enables deep-tissue excitation, while a silica-embedded photosensitizer, mesoporous DOX reservoir, and aptamer-functionalized DNA hydrogel shell collectively support targeted, stimuli-responsive therapy [28]. Folate–PEG–modified hollow mesoporous organo-silica nanoparticles (*ADCuSi-FA*) similarly illustrate multifunctional integration by embedding ultrasmall copper sulfide (*CuS*) nanodots and redox-labile drug payloads to achieve synergistic photothermal and chemotherapeutic activity [35]. Additional hybrid systems, such as glucose oxidase–gadolinium–copper sulfide (*Gd–CuS*) complexes within mesoporous silica matrices, enable catalytic therapy coupled with MRI guidance [25]. Together, these examples demonstrate how pore engineering, surface modification, and core–shell assembly empower silica platforms as versatile carriers for combined imaging and therapy.

### 3.5. Polymeric and Lipid Nanocarriers

**Figure 1:**
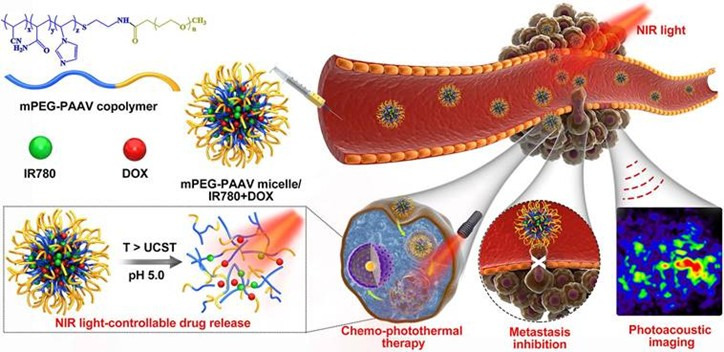
Schematic illustration of the chemo-photothermal therapy of IR780- and DOX-loaded mPEG-PAAV micelle with NIR laser-controlled drug release. Reprinted with permission from reference [31].

Polymeric and lipid-based nanocarriers offer adaptable architectures for environment-responsive drug release and enhanced tumor accumulation. Thermosensitive liposomes incorporating semiconducting polymers and gambogic acid undergo near-infrared (NIR)–triggered phase transitions, enabling controlled payload release [19]. PEGylated poly(lactic-co-glycolic acid) PLGA nanoparticles support dual functionality by combining gadolinium chelation for MRI with surface peptide modification for tumor targeting [23]. Upper critical solution temperature (UCST)–based micelles co-loaded with chemotherapeutics and NIR dyes exhibit disassembly under acidic and thermally elevated conditions, enabling spatially selective drug activation. An amphiphilic polymer—poly(ethylene glycol)-b-poly(acrylamide-co-acrylonitrile-co-vinylimi dazole) (mPEG-PAAV)—was synthesized to achieve NIR-controlled drug release; micelles formulated from this copolymer were co-loaded with DOX and IR780, as shown in Figure 1 [31]. Additionally, nitric oxide–releasing polymeric nanostructures integrate photothermal activation with hypoxia alleviation and immune modulation [36]. These systems reflect how polymer and lipid engineering enable tunable release kinetics, targeted delivery, and multifunctional performance under tumor-specific conditions.

### 3.6. Polydopamine and Hybrid Shell Architectures

Polydopamine (PDA), inspired by mussel adhesive proteins, functions as a versatile coating material that provides photothermal activity, surface reactivity, and environmental responsiveness. In systems such as *UCNP@SiO_2_-MB@PDA*—comprising an upconversion nanoparticle (UCNP) core, silica layer, methylene blue photosensitizer, and final PDA coating—PDA enhances structural stability and supports NIR-triggered therapeutic activation [16]. In bismuth vanadate/magnetite@PDA (*BiVO_4_/Fe_3_O_4_@PDA*) supra particles, PDA improves thermal conversion efficiency and stabilizes hybrid architectures [29]. Mesoporous PDA nanocarriers also act as pH-sensitive gatekeepers, enabling controlled drug release in acidic tumor microenvironments [30]. Collectively, these examples underscore PDA’s utility as a biodegradable, multifunctional shell that reinforces nanostructures and enables responsive drug delivery.

### 3.7. Biomimetic and Biohybrid Constructs

Biomimetic platforms integrate biological components to enhance immune evasion, targeting precision, and biocompatibility. Microbial–synthetic hybrids, such as lipid nanoparticles functionalized with *Bifidobacterium*, demonstrate cross-domain engineering to improve tumor colonization and immunogenic modulation [22,65]. Cell membrane-coated metallacages leverage homotypic targeting properties and natural immune escape pathways to enhance delivery efficiency [34]. Engineered *E. coli* cloaked with PDA and loaded with DOX utilize microbial vesicle interfaces to promote ultrasound-enhanced intratumoral penetration [38]. These approaches highlight the therapeutic promise of biologically inspired cloaking strategies and microbial interfaces in advancing nanotheranostics platforms.

### 3.8. Molecular and Supramolecular Agents

Molecular and supramolecular constructs operate without inorganic scaffolds, enabling precise, stimulus-responsive behaviors through rational chemical design. Donor–acceptor–donor conjugates can self-assemble into optoelectronic nanoparticles suitable for combined imaging and therapy [15]. Redox-activated ferrocene–disulfide photosensitizers [37], nitro reductase-sensitive benzo phenothiazine pro-photosensitizers [41], and aggregation-induced emission (AIE) molecules with organelle-targeting capabilities [66,67] exemplify responsive chemistries designed for spatiotemporally controlled activation. These systems demonstrate how supramolecular design principles can yield potent, targeted therapeutic agents with finely tuned activation profiles.

### 3.9. Unifying Design Principles

**Scheme 1:**
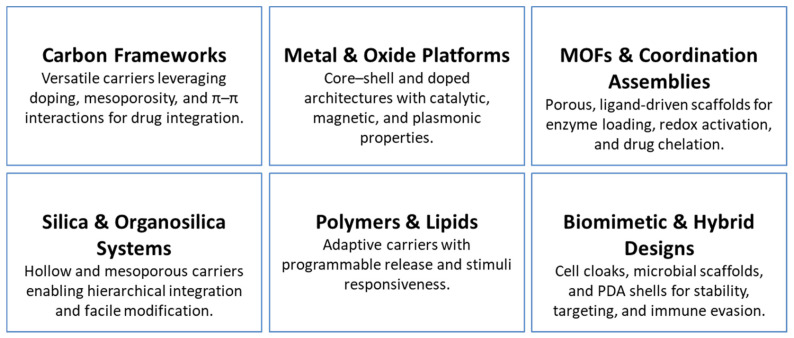
Design Strategies for Multifunctional Nanoplatforms. High-level summary of nanomaterial platforms used in synergistic tumor imaging and therapy. Designs span carbon frameworks, metals/oxides, MOFs, silica scaffolds, polymers/lipids, and biomimetic or hybrid systems, each offering distinct structural features to integrate diagnostics and therapeutics. Representative refs: [14,17,18,20,23,24,34,35].

Across this diverse landscape of nanomaterial platforms, several convergent design principles emerge. Core–shell and hollow structures enable spatially and temporally controlled drug release. Doping and defect engineering modulate redox activity, photo reactivity, and biodegradation kinetics. MOFs and coordination complexes offer modular porosity and catalytic functionality. PDA coatings provide biocompatibility alongside pH-responsive gating. Biomimetic cloaks enhance immune evasion and targeting specificity. Molecular and supramolecular systems deliver finely tuned, stimulus-triggered activation. Together, these strategies—summarized in Table 1 and depicted in Scheme 1—form a modular and rational framework for constructing multifunctional nanoplatforms optimized for integrated imaging and therapeutic precision in cancer nanomedicine.

## 4. Mechanistic Pathways Driving Nano-System Function

### 4.1. Tumor Microenvironment (TME) Responsiveness

Nano systems are frequently engineered to leverage the unique biochemical features of the TME, enabling localized activation while minimizing systemic toxicity. Acidic pH acts as a primary trigger, inducing structural disassembly in carriers such as mesoporous polydopamine, which undergoes protonation and glutathione GSH-mediated degradation to enable controlled drug release and pH-sensitive imaging [42]. Similarly, ZIF-8 frameworks exhibit acid-triggered breakdown, releasing *Zn^2+^* ions that activate aggregation-induced emission (AIE), providing real-time diagnostic feedback [39].

Redox-responsive systems offer additional layers of specificity. *MoO_x_*-based nanoplatforms deplete intracellular glutathione while simultaneously generating singlet oxygen (*^1^O_2_*) and hydroxyl radicals (•OH), thereby enhancing both photodynamic and chemo dynamic therapeutic effects [40]. More complex constructs integrate multiple TME cues: Cu-based nanoplatforms inhibit catalase activity to elevate *H_2_O_2_* concentrations, promoting Fenton-like radical generation, while embedded ultrasmall *CuS* nanodots enable enhanced NIR-II photothermal response [35]. Hypoxia-responsive mechanisms further refine spatial selectivity, exemplified by nitro reductase-activated photosensitizers capable of supporting oxygen-independent Type I photodynamic therapy [41]. Collectively, these mechanisms illustrate how TME-responsive engineering enables precise, context-specific therapeutic activation.

### 4.2. Optical Absorption, Emission, and Energy Transfer

Light-driven processes in nano systems rely on engineered strategies that amplify optical absorption, intersystem crossing, and energy transfer efficiency. Heavy-atom substitution, as demonstrated with iodine-modified aza-BODIPY, enhances intersystem crossing and singlet oxygen production while achieving a photothermal conversion efficiency (PCE) of 34.8% [68]. Spectral coupling approaches—such as pairing UCNPs that emit at 660 nm with methylene blue photosensitizers—facilitate efficient NIR-triggered photodynamic therapy [28].

Z-scheme heterojunctions, including *MoS_2_–Ti_3_C_2_* composites, enable directional charge separation and substantial superoxide (*O_2_*^−^•) generation, achieving a PCE of 59.1% under single-wavelength excitation [69]. Aggregation-induced emission (AIE) photosensitizers further enhance optical responsiveness through high quantum yield and targeted organelle localization, supporting precise mitochondrial photoactivation [67]. These optical enhancements underscore how nanoscale photo physics can be tuned to improve imaging contrast and therapeutic potency.

### 4.3. Photothermal Conversion and Hyperthermia

Photothermal conversion efficiency (PCE) is a key determinant of nano system performance, governing both heat generation and the magnitude of downstream biological effects. Reported PCE values vary across material classes, reflecting differences in composition and structural design: polymeric IR780 micelles (23.8%) [36], mesoporous polydopamine (45.6%) [30], carbon dots (54.9%) [70], *MoO_x_* hybrids (51.5%) [40], *Cu*-based catalytic platforms (57.45%) [71], and *MoS_2_–Ti_3_C_2_* heterojunctions (59.1%) [69]. These efficiencies arise from strategies such as defect engineering, optimized light absorption, and heterojunction-mediated charge dynamics.

Beyond direct thermal ablation, photothermal energy plays a multifaceted mechanistic role. It can enhance catalytic activity, increase vascular permeability, and promote drug release. *CuS* nanodots exemplify this multifunctionality by contributing simultaneously to photothermal therapy and to Fenton-like catalytic enhancement via *H_2_O_2_* decomposition [35]. These combined thermal and catalytic mechanisms significantly strengthen therapeutic performance.

### 4.4. Reactive Oxygen and Radical Generation

Reactive oxygen species (ROS) generation represents a central therapeutic mechanism in both photonic and catalytic nano systems. Conventional photosensitizer-based constructs, such as Chlorin e6 (*Ce6*)–loaded platforms, produce singlet oxygen (*^1^O_2_*) under NIR irradiation [72], while structural enhancements—such as iodine substitution in aza-BODIPY derivatives—further increase ROS output and photothermal responsiveness [68]. Catalytic nanozymes extend this capability beyond light-dependent activation. Platinum-integrated MOFs catalyze the decomposition of *H_2_O_2_* to generate *O_2_*, alleviating tumor hypoxia and sustaining photodynamic activity [73]. Iridium oxide (IrO_X_) nanozymes exhibit peroxidase- and catalase-mimetic functions, enabling simultaneous GSH depletion and hydroxyl radical (•OH) production [13]. Transition metal catalysts, including *Fe, Cu, Mn, and Co*, drive Fenton and Fenton-like reactions that induce oxidative stress and ferroptosis via lipid peroxidation [33,35,74].

Gas-releasing agents introduce an additional dimension of reactivity. Nitric oxide (*NO*) donors release *NO* upon thermal stimulation [36], while Mn carbonyl complexes liberate *CO* in response to *H_2_O_2_* or NIR irradiation, concurrently producing *Mn^2+^* ions for MRI contrast enhancement [75]. Together, these photonic–catalytic systems integrate TME responsiveness with ROS and gas generation to amplify oxidative damage and achieve selective tumor ablation.

### 4.5. Targeting and Localization Mechanisms

Targeting specificity in nano systems arises from a combination of ligand-directed interactions, biomimetic interfaces, and organelle-level localization strategies. Hyaluronic acid (HA) binds CD44 receptors frequently overexpressed in tumor cells, facilitating receptor-mediated endocytosis [76]. Arginine–Glycine–Aspartic acid (RGD) peptides engage α_vβ_3_ integrins to enhance targeting of tumor vasculature and stromal components [56]. Subcellular targeting strategies further refine therapeutic precision; for example, triphenyl phosphonium (TPP)-modified *MoS_2_–Ti_3_C_2_* heterojunctions localize to mitochondria, intensifying oxidative damage at the organelle level [69].

**Figure 2:**
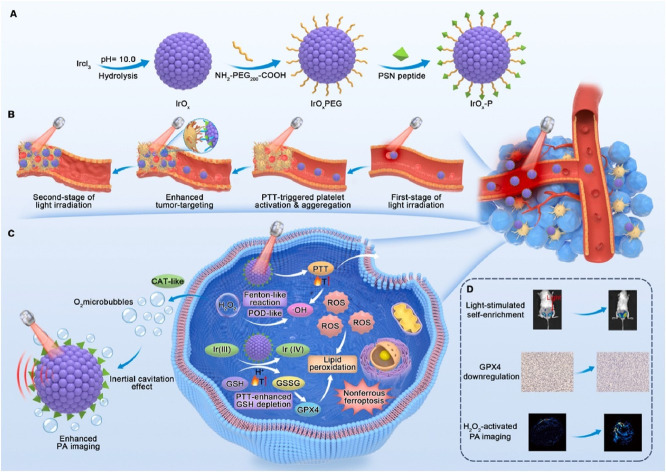
Schematic design and multimodal theranostic mechanism of IrOx-P nanoparticles. (**A**) Synthesis and functionalization process of IrOx-P nanoparticles. IrOx nanoparticles were synthesized via a hydrolysis method, followed by PEG modification and conjugation with P-selectin-targeting peptides (PSN) to form IrOx-P. The resulting nanoparticles exhibit excellent photothermal properties, enzymatic activities, and tumor-targeting capabilities. (**B**) Photothermal irradiation induces vascular endothelial damage at the tumor site, which triggers the recruitment of activated platelets. These activated platelets highly express P-selectin, enabling specific binding to the PSN conjugated on the surface of IrOx-P nanoparticles. This interaction mediates secondary enrichment of IrOx-P at the irradiated site, thereby enhancing local accumulation and subsequent therapeutic efficacy. (**C**) Schematic diagram illustrating the dual-responsive catalytic mechanism of IrOx-P for synergistic CDT/ferroptosis and PA imaging. (**D**) Representative images of light-stimulated self-enrichment, GPX4 downregulation and H_2_O_2_-acitivated PA imaging. Reprinted with permission from reference [13].

Biomimetic approaches leverage natural interfaces for enhanced homotypic recognition and immune evasion. Cancer cell membrane-coated metallacages replicate tumor antigenic profiles, enabling improved tumor-specific accumulation and prolonged circulation [34]. Bacterial systems provide a distinct modality: engineered *E. coli* selectively colonize hypoxic tumor niches, where gas vesicles facilitate ultrasound-guided cavitation [38]. Additionally, platelet membrane–interfaced IrO_X_ nanozymes, as illustrated in Figure 2, exploit P-selectin binding to self-enrich within tumor vasculature, thereby enhancing catalytic and photothermal performance [13].

### 4.6. Integrated Multifunctional Synergies

The most advanced nanoplatforms integrate multiple mechanistic components into cohesive, self-reinforcing therapeutic systems. *MoO_x_*-based hybrids illustrate this principle by combining NIR-triggered photothermal activity (PCE = 51.5%), oxygen generation, GSH depletion, and dual ROS production within a single platform [40]. *Fe*-doped sulfide nanozymes similarly unify NIR-II photothermal responsiveness with charge-transfer-enhanced Fenton catalysis and ferroptosis induction, enabling potent oxidative damage against tumor cells [33]. Compact molecular constructs such as AIE-active agents further demonstrate the efficiency of unified design, concurrently supporting high-contrast fluorescence imaging, mitochondrial targeting, ROS production, and thermal therapy within a single molecular framework [67]. These examples highlight how integrating environmental responsiveness, optical energy transduction, catalytic activation, and subcellular targeting can produce multifunctional nano-theranostics systems with synergistic therapeutic impact.

**Scheme 2:**
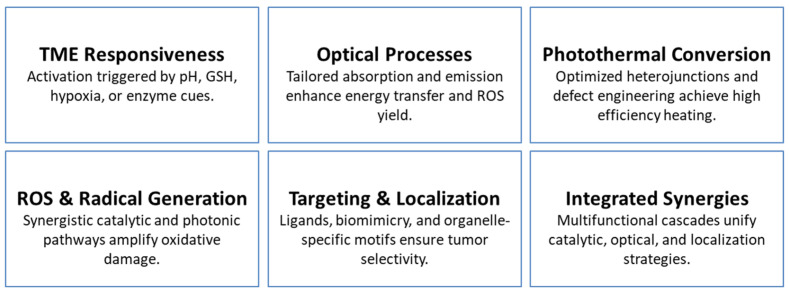
Mechanistic Pathways Driving Nanoplatform Function. Schematic overview of functional mechanisms exploited by nanotheranostics. Key domains include tumor microenvironment responsiveness, optical energy transfer, photothermal conversion, radical and ROS generation, tumor targeting, and integrated multifunctional synergies. Representative refs: [13,28,35,39,40,67,69,70].

Taken together, this mechanistic diversity—encompassing TME-responsive activation, optical energy harvesting, photothermal and catalytic enhancement, ROS and gas release, precise targeting, and multifunctional synergy—is summarized in Table 2 and visually depicted in Scheme 2. These mechanistic frameworks collectively define the functional landscape of advanced nanotheranostics systems and provide a foundation for their continued evolution toward clinically actionable platforms.

## 5. Advances in Multimodal Tumor Imaging

### 5.1. Fluorescence Imaging from Visible to NIR-II

FL imaging has progressed from traditional small-molecule probes to sophisticated nanoscale platforms capable of deep-tissue visualization, high sensitivity, and stimulus-activated specificity. Techniques such as two-photon excitation and fluorescence lifetime imaging now allow real-time monitoring of cellular apoptosis with subcellular resolution [14,24,77]. Expansion into the NIR-II window (1000–1700 nm) has markedly enhanced penetration depth and tumor-to-background contrast, enabling applications including vascular mapping, metastatic lesion detection, and bone-targeted diagnostics [34,78,79,80,81,82]. Tumor selectivity is further strengthened by TME-responsive probes that activate under acidic, hypoxic, GSH-rich, or oxidative conditions [12,37,50,83]. In parallel, FL-based nano sensors capable of detecting oncogenic mRNA and miRNA signatures have emerged, offering molecular-level discrimination between malignant and healthy tissues [2,16,39,84]. Collectively, these advances illustrate how FL imaging now integrates stimulus responsiveness, molecular targeting, and therapeutic monitoring to support precision cancer diagnostics.

### 5.2. Photoacoustic and Optoacoustic Imaging

Photoacoustic imaging (PAI) merges optical excitation with ultrasound detection, yielding high-resolution, deep-tissue imaging with excellent spatial and temporal performance. Contrast agents derived from organic dyes, polydopamine, and conjugated small molecules have become foundational tools for imaging-guided therapy [7,15,85,86]. Recent innovations, including NIR-II PAI and multispectral optoacoustic tomography, significantly enhance lesion boundary definition and detection sensitivity [35,54,82,87]. Activatable probes responsive to tumor-associated biochemical cues—such as MMPs, nitro reductase, hydrogen sulfide (*H_2_S*), and oxygen gradients—further improve specificity and tumor-selective contrast enhancement [13,41,88,89,90]. Beyond structural imaging, PAI allows real-time monitoring of dynamic biological processes including drug release, apoptosis induction, and vascular remodeling [31,77], reinforcing its emerging role as a clinically relevant modality for integrated theranostics.

### 5.3. Magnetic Resonance Imaging and Contrast Innovation

MRI remains a cornerstone of clinical diagnostics due to its deep tissue penetration and high spatial resolution. Nanoparticles incorporating paramagnetic metals such as *Mn*, *Fe*, and *Gd* provide strong T_1_ and T_2_ contrast enhancement [18,59,75,91,92]. Dual-mode T_1_/T_2_ nanoprobes address diagnostic ambiguity and improve lesion delineation [64,74], while activatable MRI agents responsive to pH, GSH, and redox gradients enable tumor-specific contrast modulation [5,6,32,93]. Fluorine-based (*^19^F*) MRI probes offer background-free imaging for unambiguous detection of labeled agents and simultaneous therapeutic monitoring [93,94]. Recent advances in contrast performance—including r_1_ relaxivity values reaching 89.1 mM^−1^s^−1^ [59]—significantly exceed clinical standards, reflecting heightened material sophistication. Additionally, many MRI-active nano systems now integrate therapeutic modalities such as redox regulation, oxygen generation, and photothermal or chemo dynamic activation, supporting precision-guided interventions.

### 5.4. Computed Tomography Imaging

CT benefits significantly from nano systems that incorporate high atomic number (Z) elements such as *Au*, *Pt*, *Bi*, and *W*, which provide strong X-ray attenuation and enhanced tumor contrast [17,87,95,96,97]. Many of these agents also function as photothermal transducers or radiosensitizers, supporting synergistic imaging and therapeutic applications. Multimodal integration with MRI, FL, and PAI strengthens diagnostic performance by combining structural, functional, and molecular information [29,42,73,98]. Representative examples include *Pt-tipped Au@ZIF-8* nanorods, which offer CT imaging capability, plasmon-enhanced phototherapy, and pH/GSH-responsive drug release [17], as well as *BiVO_4_/Fe_3_O_4_@PDA* hybrid particles that combine CT, MRI, and PAI for guided therapeutic intervention [29]. Some platforms also catalyze ROS generation and relieve hypoxia, further enhancing therapeutic outcomes [73]. Tri-modal constructs such as DMCR nanoprobes integrate CT, MRI, and FL imaging with targeted chemo-photothermal therapy, highlighting the translational potential of CT-compatible nanotheranostics [42].

### 5.5. Ultrasound and Hybrid Imaging

US imaging continues to advance through nanoscale contrast agents engineered to enhance acoustic responsiveness and enable real-time, image-guided therapy. Phase-transition nanodroplets—such as those containing perfluoro hexane (PFH)—undergo acoustic droplet vaporization, improving image contrast and supporting high-intensity focused ultrasound (HIFU)–mediated tumor ablation [4,30,63]. Bubble-generating systems further amplify cavitation effects and enable spatially precise drug release. More recent developments include genetically engineered bacteria capable of producing gas vesicles, which function as endogenous US contrast agents with intrinsic tumor-targeting capacity. These microbial platforms preferentially colonize hypoxic tumor regions and support multimodal imaging, including MRI and PAI [22,38,99]. As a real-time, cost-effective, and clinically accessible modality, ultrasound continues to expand its role in theranostic integration.

### 5.6. Multimodal Integration and Functional Diagnostics

A defining direction in contemporary cancer imaging is the development of nanoplatforms that integrate multiple imaging modalities to deliver comprehensive anatomical, molecular, and functional information. Tri- and tetra-modal constructs incorporating MRI, CT, FL, PAI, and US provide multidimensional tumor visualization by capturing high-resolution structural features alongside dynamic microenvironmental changes [29,42,48,73,100]. Systems fusing NIR-II fluorescence with MRI and CT enable deep-tissue profiling with enhanced spatial fidelity [73,98]. These platforms also support real-time monitoring of key biological processes including apoptosis [77], hypoxia [41,60], vascular perfusion [25,31], and redox stress [3,12,37,50,83,90]. Activatable imaging agents that respond to pH, GSH, oxygen tension, and endogenous gas transmitters further refine tumor selectivity by suppressing off-target signals. Additionally, RNA-responsive nanoprobes capable of detecting oncogenic mRNA and miRNA signatures through FL or PAI outputs enhance molecular precision in diagnostic workflows [2,16,39,84]. By seamlessly combining diagnostic and therapeutic functions, these multimodal constructs enable adaptive, feedback-driven treatment strategies.

**Scheme 3:**
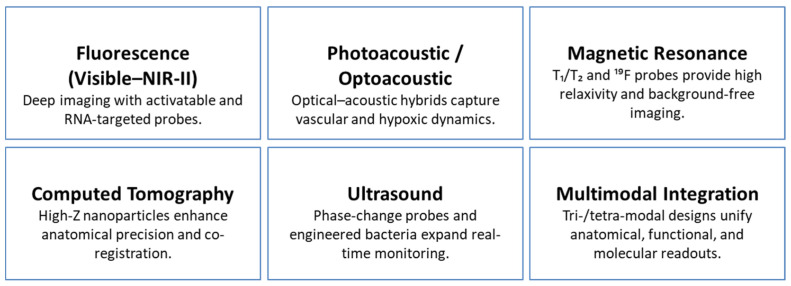
Synergistic Imaging Across Modalities. Summary of imaging strategies integrated into nanoplatforms. Modalities span fluorescence (visible–NIR-II), photoacoustic, MRI, CT, ultrasound, and multimodal hybrids. Together, these provide complementary anatomical, functional, and molecular resolution for precision tumor profiling. Representative refs: [14,18,22,29,42,73,78,94,98].

Collectively, advances in nanotheranostics imaging illustrate a clear progression—from single-modality contrast agents to multifunctional, activatable platforms capable of real-time, targeted, and integrated diagnostics. By merging deep-penetrating modalities such as MRI, CT, PAI, and NIR-II FL with responsive functional outputs, these systems support comprehensive tumor characterization at both anatomical and molecular levels. The incorporation of targeting ligands, biomimetic interfaces, and TME-specific activation mechanisms further improves accuracy and facilitates therapeutic co-delivery. This evolving diagnostic paradigm—summarized in Table 3 and depicted in Scheme 3—positions multimodal nanoplatforms as next-generation tools for early detection, intraoperative guidance, and dynamic therapy monitoring within precision oncology.

## 6. Synergistic Therapeutic Modalities in Nanomedicine

### 6.1. Photothermal and Photodynamic Therapy as a Central Hub

PTT and PDT serve as central pillars of nanotheranostics, with their synergistic combination consistently outperforming either modality alone. PTT produces localized hyperthermia, while PDT generates ROS; together, they enable spatially confined, amplified cytotoxicity. Early systems such as sulfur–nitrogen–doped carbon dots (S,N-CDs) demonstrated this synergy through concurrent ROS generation, photothermal conversion, and tumor-selective fluorescence imaging [14].

More advanced constructs—including *HA-TiO_2_@MWCNTs/HMME* [76] and iodinated aza-BODIPY nanoparticles [68]—achieve tumor-targeted delivery, dual-mode phototherapy, and integrated diagnostic functionality. To address hypoxia-associated limitations in PDT, catalytic platforms capable of in situ oxygen generation have been developed, such as catalase-loaded gold nano stars [56] and *MONs@PDA-ICG* nanosheets [6]. These innovations position PTT/PDT as a central therapeutic axis around which multifunctional, TME-responsive nanoplatforms are increasingly designed.

### 6.2. Chemo–Photo Synergies

Combining chemotherapy with phototherapies enhances therapeutic selectivity while reducing systemic toxicity through spatiotemporally controlled drug release. The DOX–Pt-tipped Au@ZIF-8 system exemplifies this synergy by enabling laser-triggered activation of chemotherapy, PDT, and PTT through plasmon-enhanced catalysis and oxygen modulation [17]. Enzyme-responsive platforms such as MMP-cleavable gold nanoparticles selectively aggregate within protease-rich tumors, improving NIR absorption and facilitating heat-enhanced DOX release [89].

Thermo- and pH-responsive platforms—including PFH-loaded PDA nanoparticles [30] and polymeric micelles [31]—enable light-triggered therapeutic release with concurrent imaging. Multifunctional constructs such as DMCR nanoprobes integrate GSH/pH-responsive DOX release with CT/MR/FL imaging and PTT [42]. Targeted nano carriers such as artemisinin-loaded RGD-IBA systems leverage receptor-mediated uptake and NIR-triggered co-release of chemotherapeutic and phototherapeutic agents [102]. These co-activation strategies transform chemotherapy into a synergistic, image-guided modality.

### 6.3. Catalytic Pathways: CDT and Ferroptosis

Chemo dynamic therapy (CDT) and ferroptosis amplify phototherapeutic outcomes through ROS generation and lipid peroxidation. PBAM MOFs first established this synergy by integrating *Mn^2+^-*driven Fenton-like catalysis with Prussian blue-based photothermal effects [88]. *Fe*-doped *MoO_x_* nanowires extend this paradigm via MRI-guided CDT enhanced by acidic pH and photothermal heating [18]. In *Cu/CC* nanoparticles, NIR-induced hyperthermia promotes Fenton catalysis and GSH depletion, intensifying oxidative stress [83].

Ferroptosis—an iron-dependent, lipid-peroxidation-driven cell death pathway—is increasingly incorporated into nanoplatforms. *Fe^3+^*-DOX liposomes release Fe^2+^ through GSH reduction, generating hydroxyl radicals and triggering ferroptosis alongside apoptosis [1]. Multifunctional systems such as *SRF@Hb-Ce6* orchestrate PDT, ferroptosis, and immune activation via sorafenib delivery and IFN-γ sensitization [103]. IrO_X_-P nanozymes exemplify high-level integration by combining PTT, CDT, and ferroptosis with platelet-mediated tumor targeting and hyperthermia-driven oxygen generation for imaging-guided tumor suppression [13]. These catalytic strategies demonstrate how CDT and ferroptosis overcome therapeutic resistance and enhance ROS-dependent cytotoxicity.

### 6.4. Immunotherapy Coupled with Local Modalities

Phototherapies inherently induce immunogenic cell death (ICD), releasing tumor antigens that can be harnessed to trigger systemic antitumor immunity. Integrating immunoadjuvants or checkpoint inhibitors within nanoplatforms magnifies this immunological impact. *ICG-CpG@MOF*, for example, couples photo-induced antigen release with CpG-mediated TLR9 activation, converting immune-cold tumors into responsive phenotypes [100]. FYH-PDA-DOX enables pH/NIR-triggered chemotherapy and PTT to induce ICD, and in combination with PD-L1 blockade, markedly increases T-cell infiltration and survival [104].

Biomimetic constructs such as metallacages nanoparticles (MCNPs) leverage tumor-cell-membrane cloaking for immune evasion and dendritic-cell activation through PDT/PTT-induced ICD [34]. The *m@MTT* nano heterojunction integrates mitochondrial ROS generation with CpG delivery to suppress primary and metastatic tumors under 808 nm irradiation [69]. More complex systems such as (*AP*)*FeNSs* combine hypoxia-activated chemotherapy (AQ4N), macrophage reprogramming, and ROS-induced ICD for multi-layered immuno-photochemotherapy [53]. These approaches demonstrate how phototherapy can serve as an immunological primer, enabling local treatments to drive systemic tumor control.

### 6.5. Radiotherapy and Acoustic Synergies

Radiotherapy (RT) and ultrasound-based modalities extend nanotherapeutic reach to deep or hypoxic tumors that challenge purely optical approaches. PEGylated *W-doped TiO_2_* nanoparticles integrate NIR-II PTT with RT, enabling thermo-radiotherapy with CT/PAI guidance [87]. *BiVO_4_/Fe_3_O_4_@PDA* super particles combine radio sensitization with tri-modal imaging (CT/PA/MR) for synergistic tumor inhibition in oral cancer models [29].

Ultrasound-driven platforms introduce additional flexibility. Dibenzo cyclooctyne-modified zinc(II)-phthalocyanine-loaded (*DBCO-ZnPc-LP*) liposomes employ click chemistry for tumor targeting, while ZnPc converts NIR light into heat and shockwaves for combined thermal and mechanical ablation [105]. *F3-PLGA@MB/Gd* agents support sonodynamic therapy (SDT), HIFU ablation, and imaging through methylene blue and Gd co-delivery [23].

Biohybrid constructs such as *GVs-E. coli* expand this capability through tumor-selective colonization, ultrasound-guided imaging, cavitation enhancement, and focused ultrasound ablation surgery (FUAS) [38]. Together, these systems showcase the potential of non-optical stimuli—including ionizing radiation and ultrasound—to broaden clinical applicability.

### 6.6. Gas, Starvation, and Metabolic Therapies

Targeting tumor metabolism and exploiting stress pathways provide powerful means of amplifying oxidative therapies. GOx-loaded platforms disrupt glycolysis while converting glucose into *H_2_O_2_*, strengthening CDT and PDT effects. Complex designs such as *GOx@NPs* using pillar [6]arene-hosted Azo-G enable hypoxia-, pH-, and esterase-responsive activation, integrating starvation therapy with oxidative and chemotherapeutic functions via intracellular logic elements [106].

Hybrid constructs such as *AUC-GOx/Cel* synergistically induce mitochondrial damage through celastrol while releasing *Cu^+^* from *CuS* degradation to accelerate Fenton catalysis, enabling feedback-enhanced CDT with real-time imaging [12]. Gas-releasing nanoplatforms add further therapeutic versatility: P1-CapNO nanoparticles trigger *NO* release under 808 nm irradiation to enhance PTT and mitigate PDT resistance [36], while *MnCO@CuS* constructs combine *CO* therapy with *Mn^2+^*-based MRI via H_2_O_2_-triggered decomposition [75]. Hypoxia-adaptive *H_2_-*releasing systems such as *MA@NHC* integrate melanin-mediated PTT with controlled *H_2_* delivery, promoting tumor ablation while limiting inflammation [107]. These strategies emphasize the therapeutic potential of metabolic disruption, redox modulation, and gas signaling.

### 6.7. Theranostic Integration

Emerging nanoplatforms increasingly combine diagnostic and therapeutic capabilities to enable real-time feedback, adaptive dosing, and dynamic treatment control. The PEGylated *DCNP@DMSN-MoO_x_* platform exemplifies this integration by uniting CDT and PTT with NIR-II fluorescence, CT, and MRI guidance under 808 nm activation. *MoO_x_* mediates singlet oxygen production, GSH depletion, and oxygen evolution, coupling imaging feedback with therapeutic action [98].

**Figure 3:**
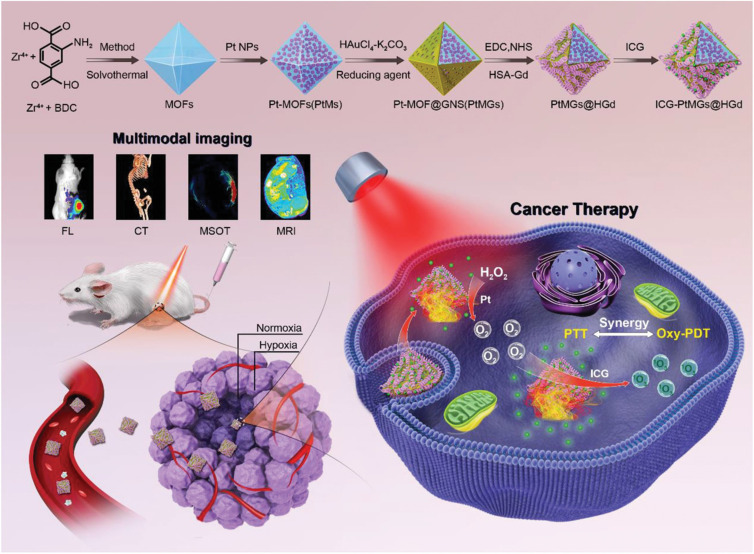
Schematic illustration of the *ICG—PtMGs@HGd* nanoplatforms as *H_2_O_2_—*driven oxygenator for FL/MOST/CT/MRI multimodal imaging guided enhanced PDT and PTT synergistic therapy in a solid tumor. Reprinted with permission from reference [73].

Multimodal constructs such as DUPM nanoparticles integrate chemotherapy, PDT, PTT, and hypoxia alleviation within a PDA-coated UiO-66 scaffold, supporting pH/GSH-responsive release, 51.5% photothermal efficiency, and >94% in vivo tumor suppression under PAI guidance [40]. *ICG-PtMGs@HGd* platforms combine oxygen self-generation with FL/MSOT/CT/MRI imaging and synergistic phototherapy, as shown in Figure 3 [73]. Additional systems—such as MSCO-PEG heterojunctions [108], BBT-IR/Se-MN radiosensitizers [3], and ZGC-coated MSN luminescent agents [58]—demonstrate how embedded sensing, activation, and imaging modalities are merging into unified theranostic frameworks.

**Scheme 4:**
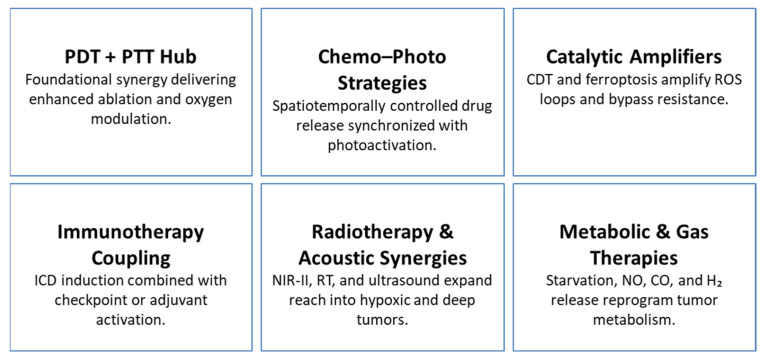
Synergistic Therapeutic Modalities in Nanotheranostics. Conceptual overview of therapeutic combinations enabled by nanoplatforms. Synergies include PDT+PTT, chemo-photo strategies, catalytic amplifiers (CDT/ferroptosis), immunotherapy coupling, radiotherapy, and acoustic integration, and metabolic or gas-based therapies. Representative refs: [12,13,14,17,23,36,75,87,100].

As a whole, the field has progressed from simple PDT/PTT combinations to sophisticated platforms integrating chemotherapy, catalytic therapy, immunotherapy, radiotherapy, ultrasound modalities, metabolic disruption, gas therapy, and multimodal imaging. This convergence—summarized in Table 4 and Scheme 4—establishes synergy, rather than monotherapy potency, as the hallmark of next-generation nanotheranostics.

## 7. Preclinical Outcomes and Translational Readiness

### 7.1. Potent Tumor Inhibition and Complete Ablation

Preclinical studies consistently show that advanced nanoplatforms can achieve complete tumor eradication and prevent recurrence across diverse models. NM-NPs produced full tumor elimination in murine systems [78], while DAA nanoparticles induced complete regression without relapse [109]. DOX/PPy-ELP-F3 nanoparticles similarly ablated tumors without detectable systemic toxicity [9]. Several systems also demonstrated efficacy against metastatic disease: mPEG-PAAV micelles eradicated primary breast tumors while suppressing pulmonary metastases [31], and Anti-tumor and Bone-repair Integrated biomineralizing Nano systems (ABI Nys) combined photothermal and immune therapies to ablate bone metastases and inhibit tumor-induced osteolysis [82]. Collectively, these outcomes underscore the potential of rationally engineered nano platforms to address both primary and metastatic disease with high therapeutic efficacy.

### 7.2. Synergistic and Multimodal Therapeutic Mechanisms

Highly effective nanotherapeutics rely on synergistic, multimodal mechanisms rather than single-agent cytotoxicity. Platforms such as *DOX–Pt-tipped Au@ZIF-8* [17] and *IR780/PTX/FHSV* micelles [110] significantly enhanced tumor suppression by simultaneously activating chemotherapy and phototherapy. Cascade-based constructs provide further amplification: *Fe^3+^–*DOX nanoliposomes combined apoptosis with ferroptosis [1], FE-T nanoparticles induced mitochondrial dysfunction alongside ICD [81], and *Cu/CC* assemblies enabled trimodal ROS-mediated therapy involving photothermal, chemo dynamic, and catalytic processes [83]. The IrO_X_-P nanozyme exemplifies fully integrated synergy, uniting PTT, CDT, and ferroptosis into a self-reinforcing therapeutic loop [13]. These findings reinforce that multimodal synergy is a key driver of translational success in nanomedicine.

### 7.3. Immune Activation and Systemic Responses

Many preclinical nanoplatforms elicit potent systemic antitumor immunity by inducing immunogenic cell death (ICD) and supporting immune activation. Fucoidan-coated, Doxorubicin-loaded, mesoporous Polydopamine (FYH-PDA-DOX) nanoparticles triggered strong T-cell responses through chemo–photothermal–immunotherapy [104], while *TAPP-GCP@TCPP@BSA* induced ICD, enhanced antigen presentation, and synergized with PD-L1 blockade to inhibit distant tumor growth [111]. Albumin-based nanocomposites similarly activated immune responses and checkpoint inhibition, producing regression of both primary and metastatic TNBC lesions [112]. These results demonstrate that nanoplatforms can function as immune sensitizers, transforming local therapy into systemic tumor control.

### 7.4. Tumor Microenvironment Responsiveness and Resistance Avoidance

TME responsiveness has emerged as a critical strategy for overcoming therapeutic resistance. *H_2_S-*activated MOFs selectively suppressed orthotopic colon tumors while minimizing systemic toxicity [90]. P1-CapNO nanoparticles released nitric oxide upon NIR activation, alleviating hypoxia-associated resistance [36]. *AUC-GOx/Cel* nano factories used dual *H_2_O_2_-*generating pathways to amplify CDT through catalytic feedback [12]. By aligning therapeutic activation with tumor-specific biochemical cues—including redox stress, acidity, and enzyme overexpression—these systems enhance precision targeting and help circumvent both intrinsic and acquired resistance mechanisms.

### 7.5. Imaging-Guided Precision and Theranostic Integration

Theranostic platforms improve translational readiness by pairing therapeutic action with real-time imaging guidance. *UCNP@SiO_2_-MB@PDA* supported combined PDT/PTT with NIR imaging assistance [16], while *PC61BA-*(*Gd-DO3A*)*/HSA* nanoparticles provided high-relaxivity MRI contrast for accurate tumor localization [59]. *MnCO@CuS* constructs enabled MRI-guided multimodal therapy by integrating gas release, photothermal ablation, and chemo dynamic activation [75]. Carboxymethyl cellulose (CMC) nanofibers combined activatable imaging with redox-triggered CDT for deep-seated tumor treatment [94]. These systems transform imaging from a passive diagnostic tool into a mechanism for precise, temporally controlled therapy.

### 7.6. Safety, Biocompatibility, and Clinical Readiness

Biocompatibility, biodegradability, and safe clearance are essential for clinical translation. *PEGylated W-doped TiO_2_* nanoparticles exhibited negligible systemic toxicity and were completely cleared within 30 days [87]. IABN nanoparticles achieved complete tumor ablation with minimal off-target effects and demonstrated excellent biocompatibility in vivo [68]. DUPM nanomedicine maintained a 94.43% tumor inhibition rate and passed preclinical safety evaluations [40]. *PC61BA-*(*Gd-DO3A*)*/HSA* nanoparticles also showed favorable tumor accumulation without adverse effects [59]. These outcomes indicate that current generation nanotherapeutics are approaching the key translational benchmarks required for eventual clinical use.

### 7.7. Overall Translational Outlook

**Scheme 5:**
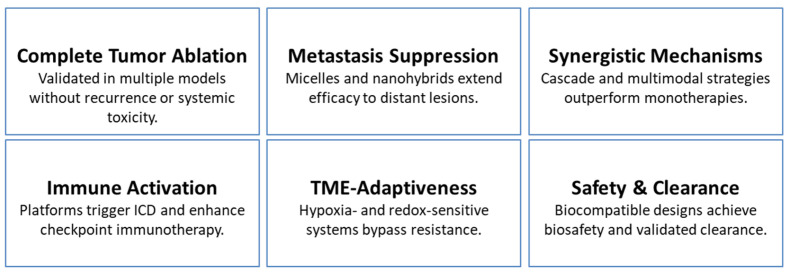
Translational Significance of In Vivo Outcomes. Visual synthesis of preclinical outcomes highlighting translational readiness. Nanoplatforms demonstrate complete ablation, metastasis suppression, multimodal synergy, immune activation, tumor microenvironment adaptability, and validated biosafety and clearance profiles. Representative refs: [13,17,36,40,59,78,81,82,112].

Collectively, these preclinical findings affirm the clinical promise of multifunctional nanoplatforms. By integrating potent tumor ablation, metastatic suppression, immune modulation, TME responsiveness, real-time imaging, and demonstrated biosafety, these systems address core challenges in oncology. Their dual therapeutic–diagnostic capabilities support a new paradigm of precision nanomedicine. These advances—summarized in Table 5 and illustrated in Scheme 5—position multifunctional nano systems as compelling candidates for next-generation cancer treatment, offering comprehensive approaches to resistance, recurrence, and systemic disease management.

## 8. Persistent Limitations of Nano-Theranostic Platforms

Despite substantial advancements, several persistent limitations continue to hinder the clinical translation of multifunctional nanotheranostics systems.

### 8.1. Biological and Microenvironmental Variability

Tumor heterogeneity remains a major challenge for achieving consistent in vivo performance. Although constructs such as mPEG-PAAV micelles have shown efficacy against both primary and metastatic lesions [31], their dependence on the enhanced permeability and retention (EPR) effect introduces significant variability across tumor types, progression stages, and vascular phenotypes [9,13]. Metabolic interventions—including GOx-mediated starvation therapy—can potentiate oxidative stress and enhance combinatorial treatment outcomes [12,106], but their overall effectiveness is often constrained by the metabolic plasticity of tumor cells and fluctuations in nutrient availability. Additionally, the tumor microenvironment (TME) evolves dynamically during disease progression and treatment, with shifting gradients of hypoxia, acidosis, and redox balance that may progressively diminish the responsiveness of TME-activated therapies [12,39,40,42]. These biological variations complicate both prediction of therapeutic outcomes and optimization of treatment regimens.

### 8.2. Complexity Versus Clinical Practicality

Although highly integrated systems—such as DUPM nanomedicine [40] and multimodal imaging-guided platforms [42,73,98]—demonstrate impressive multifunctionality, this complexity often presents considerable barriers to clinical implementation. Multi-component architecture typically require intricate synthesis, precise assembly, and tight quality-control parameters, increasing the risk of batch-to-batch variability and limiting manufacturing scalability. Materials with strong preclinical performance—such as MOFs and semiconductor heterojunctions [24,33,69]—may lack standardized, GMP-compatible production methods, complicating regulatory approval and industrial translation. These factors underscore an ongoing tension between maximizing functionality and ensuring manufacturability, reproducibility, and cost-effectiveness.

### 8.3. Safety, Clearance, and Biocompatibility

While several nanoplatforms exhibit promising biosafety profiles—such as the complete systemic elimination of *W-doped TiO_2_* nanoparticles within 30 days [87]—long-term biodistribution, potential immune activation, and incomplete clearance remain insufficiently characterized for many systems incorporating inorganic components (e.g., *Gd*, *Mn*, *Au*) [18,59,75]. Biomimetic cloaking strategies, including tumor-cell-membrane and bacteria-derived coatings [34,38], can improve immune evasion and targeting specificity; however, their reliance on autologous or patient-derived materials introduces practical challenges related to standardization, storage stability, manufacturing consistency, and regulatory approval. These considerations highlight the need for comprehensive evaluation of biocompatibility, immunogenicity, and long-term retention under conditions that accurately reflect clinical use.

## 9. Future Priorities for Clinical Translation

### 9.1. Adaptive and Feedback-Driven Platforms

Theranostic systems capable of real-time monitoring and adaptive therapeutic control represent a major direction for improving treatment precision and safety. Constructs such as *UCNP@SiO_2_-MB@PDA* [16] and *MnCO@CuS* [75] demonstrate how integrated imaging modalities can guide spatially resolved interventions. Future platform design will benefit from closed-loop architectures in which diagnostic feedback actively adjusts therapeutic output, allowing dynamic modulation in response to evolving tumor microenvironmental cues and minimizing off-target damage.

### 9.2. Streamlined Multifunctionality

Simplifying multifunctional designs without compromising performance is critical for clinical scalability. Single-molecule AIE systems such as TPETTBI [67], which unify fluorescence imaging, organelle targeting, ROS generation, and photothermal activity, illustrate how molecular-level integration can reduce structural complexity. Similarly, defect-engineered oxides and doped sulfides [18,33,69] provide intrinsic multifunctionality through precise compositional tuning, decreasing reliance on multi-component hybrid assemblies and improving reproducibility across manufacturing batches.

### 9.3. Integration with Immunotherapy

Nanoplatforms capable of inducing ICD [69,100,104,111] or enhancing immune checkpoint blockade [34,104,112] offer promising avenues for alignment with established immunotherapy paradigms. Strategically embedding nano systems within validated immunotherapeutic regimens may accelerate clinical adoption by leveraging existing mechanisms of antitumor immunity and compatible clinical workflows.

### 9.4. Targeting Metastasis and Resistant Niches

Combatting metastatic dissemination and therapy-resistant tumor niches remains a priority for translational nanomedicine. Platforms capable of targeting lung [31] and bone [82] metastases provide early proof-of-concept for organ-specific delivery. Microenvironmental modulation—such as hypoxia alleviation [36] or metabolic disruption via starvation therapy [12,106]—further supports durable therapeutic responses in heterogeneous and plastic tumor ecosystems.

### 9.5. Scalability and Regulatory Readiness

Clinical translation requires platforms that can be manufactured at scale under regulatory-compliant conditions. Green synthesis methods (e.g., phloroglucinol-derived carbon dots [20]) and biodegradable carriers such as PLGA and polydopamine [23,30] offer practical, clinically aligned routes to commercialization. Comprehensive biosafety assessments—including long-term biodistribution, immune compatibility, and metabolic clearance—remain essential. Encouraging precedents from systems such as WTO [87], DUPM [40], and IABN [68] highlight the feasibility of meeting these criteria, though translation ultimately depends on validation in large-animal models and standardized, GMP-ready production processes.

## 10. Conclusions

Synergistic imaging has become a defining feature of next-generation nanotheranostics, transforming imaging from a passive diagnostic tool into an active, feedback-regulated component of therapeutic decision-making. By integrating multimodal imaging with photothermal and photodynamic therapies, catalytic amplification, immune activation, and metabolic modulation, emerging platforms achieve high levels of spatial precision and therapeutic potency in preclinical cancer models.

Despite these advances, clinical translation remains hindered by persistent challenges, including tumor heterogeneity, structural complexity, and incomplete long-term safety characterization. Addressing these limitations will require streamlined multifunctional systems, closed-loop imaging–therapy architectures, and patient-stratification frameworks grounded in molecular and microenvironmental profiling.

The convergence of synergistic imaging with systemic immunotherapy, intraoperative navigation, and adaptive therapeutic design represents a compelling frontier for the field. With continued refinement and translational focus, nanotheranostics are poised to evolve from experimental constructs into clinically impactful technologies—positioning multifunctional nano systems as core enablers of precision oncology.

### Evidence-to-Practice Roadmap for Nanotheranostics in Oncology

As the field of nanotheranostics continues to mature, bridging preclinical innovation with clinical translation has become a central challenge. The integration of diagnostic precision and multimodal therapeutic function holds transformative potential for oncology, yet translation requires systematic alignment between mechanistic insight, clinical need, and implementation feasibility. Table 6 outlines an *Evidence-to-Practice Roadmap* that synthesizes the current state of nanotheranostics platforms across major therapeutic themes—from photothermal and photodynamic hubs to catalytic and immunomodulatory systems. Each thematic section delineates the rationale behind development, mechanistic core principles, and observed preclinical outcomes, while also identifying target clinical populations and key translational priorities. By highlighting both demonstrated efficacy and unresolved gaps, this roadmap provides a structured framework for advancing nanotheranostics interventions from laboratory validation toward standardized, patient-specific clinical applications.

**How to read/use this roadmap**

- **What we know:** Columns 2–6 aggregate proven needs, mechanisms, preclinical signals, and where they fit clinically.
- **What we don’t know:** Column 8 captures gaps (e.g., **patient heterogeneity, metal fate, dosing windows, workflow standardization**).
- **Future priorities:** Column 7 converts gaps into **actionable next steps** (e.g., **closed-loop theranostics**, **biomarker-led stratification**, **simplified/GMP-feasible builds**).

## Figures and Tables

**Table 1:** Overview of Nanoplatform Design and Fabrication Strategies.

Nanomaterial/System Design & Fabrication	Examples & Structural Features	Expert Insights	Representative Refs.
Carbon-Based Nanostructures	S, N-CDs (hydrothermal); g-CQDs (acid-assisted synthesis); HMCS (PEGylated, GA-loaded); MWNT-based hybrids.	Heteroatom doping, mesoporosity, and π–π interactions enhance optical properties, loading capacity, and molecular specificity.	[14,20,26,27]
Metal & Metal Oxide Platforms	*Pt-tipped Au@ZIF-8*; Fe-doped *MoO_x_* nanowires; *Gd-MFe_3_O_4_* mesoporous nanoparticles; *MnO_2_-*coated GO/ICG & DOX systems.	Alloying, heteroatom doping, and surface-shell engineering enable tunable redox reactivity, controlled biodegradation, and enhanced catalytic/plasmonic functions.	[17,18,21,64]
MOFs & Coordination Assemblies	*Fe/Cu-MOF-199@PDA*; UiO-67 CIDF; ZIF-67-derived FCS; DOX@FL coordination networks.	MOF porosity and modular coordination chemistry support high loading capacity, redox activation, and direct assembly of therapeutic agents.	[5,24,32,33]
Silica & Organo-silica Systems	UCNJ tri-layer (core–shell–hydrogel); HMONs with ultrasmall *CuS/DSF/3-AT; GOx–Gd–CuS@MSNs*.	Silica frameworks enable hierarchical integration of multiple agents with robust porosity control and facile surface functionalization.	[25,28,35]
Polymeric & Lipid Systems	Thermosensitive liposomes (DG@TLs); PLGA@MB/Gd nanoparticles; UCST micelles (mPEG-PAAV); P1-CapNO polymers.	Polymer and lipid matrices offer tunable size, responsive release behavior, and programmable encapsulation for combination therapy.	[19,23,31,36]
PDA & Hybrid Shells	*UCNP@SiO_2_-MB@PDA; PFP@MPDA-DOX; BiVO_4_/Fe_3_O_4_@PDA* supra particle.	PDA coatings provide universal adhesion, photothermal functionality, biodegradability, and pH-responsive drug regulation.	[16,29,30]
Biomimetic & Biohybrid Platforms	*Bifidobacterium*–nanoparticle hybrids; membrane-cloaked MCNPs; engineered *E. coli*@PDA-DOX.	Microbial scaffolds and cell-membrane cloaks support homotypic targeting, immune evasion, and enhanced intratumoral accumulation.	[22,34,38,65]
Molecular & Supramolecular Agents	DPP-TPA nanoparticles; *ferrocene–disulfide PS* (*HFP-SS-Fc*); benzo phenothiazine pro-PS (BPN); AIE systems (TPETTBI, TPN-Cb).	Molecular-level engineering enables intrinsic responsiveness (AIE, redox activation, caging) without reliance on inorganic scaffolds.	[15,37,41,66,67]

**Table 2:** Overview of Mechanistic Functional Properties in Nanoplatforms.

Mechanistic Functional Properties	Examples & Explanations	Expert Insights	Representative Refs.
pH-Responsive Activation	MPDA undergoes decomposition in acidic/GSH-rich TME, enabling drug release and fluorescence activation; ZIF-8 MOFs disassemble under acidic pH, releasing *Zn^2+^* and enhancing AIE output.	Acidic TME conditions are leveraged for both structural disassembly and real-time signal amplification.	[39,42]
GSH-Responsive Redox Cycling	*MoO_x_* platforms deplete intracellular GSH while generating *^1^O_2_* and *·OH*; *Cu* nanodots—with 3-AT–mediated catalase suppression—accelerate *Cu^+^-*driven Fenton chemistry under NIR-II.	GSH depletion simultaneously weakens tumor antioxidant defenses and sustains redox-driven therapeutic cycling.	[35,40]
Hypoxia-Triggered Activation	NTR-activated pro-photosensitizers switch on fluorescence and initiate oxygen-independent Type I PDT in hypoxic regions.	Hypoxia acts as a biochemical gate, enabling selective PDT with minimal background activation.	[41]
Optical Energy Transfer	UCNJs emit 660 nm light to excite methylene blue, generating singlet oxygen.	Precise spectral coupling maximizes excitation efficiency and enhances PDT performance.	[28]
Heavy-Atom Effect for ROS	Iodine-substituted aza-BODIPY increases *^1^O_2_* yield by 1.57× compared to bromide analogs and achieves 34.8% PCE.	Heavy-atom substitution enhances intersystem crossing and boosts ROS formation.	[68]
Heterojunction Charge Transfer	*MoS_2_–Ti_3_C_2_* Z-scheme heterojunction exhibits 59.1% PCE and robust *O_2_^−^*· generation via directional charge separation.	Efficient charge separation strengthens both photothermal output and photocatalytic ROS generation.	[69]
Photothermal Conversion Efficiencies	Carbon dots (54.9%); *MoO_x_* (51.5%); *Cu^2+^* catalytic NPs (57.45%); MPDA (45.6%); polymeric IR780 micelles (23.8%).	Highest PCE values typically arise from heterojunction structures and defect-engineered nanomaterials.	[30,36,40,69,70,71]
Catalase-Like Activity	Pt-decorated constructs catalyze *H_2_O_2_* to *O_2_*; IrO_X_ nanozymes simultaneously deplete GSH and catalyze *·OH* and *O_2_* production.	Catalase-mimetic functions enhance intratumoral O_2_ availability while amplifying ROS-mediated therapy.	[13,73]
Fenton & Fenton-Like Catalysis	Fe-doped sulfides enhance electron transfer to boost ·OH generation; *CuS* nanodots combine NIR-II photothermal heating with *Cu^+^* Fenton catalysis.	Transition-metal doping narrows band gaps and accelerates catalytic reaction kinetics.	[33,35,74]
Gas Therapeutics	*NO*-releasing platforms enable thermally activated *NO* delivery (lifetime ~2 s, diffusion ~200 µm); Mn carbonyl complexes release *CO* under *H_2_O_2_*/NIR and degrade to *Mn^2+^*.	Gas transmitters expand therapeutic mechanisms beyond ROS, enabling O_2_-independent modalities.	[36,75]
Targeting Mechanisms	HA for CD44 recognition; c(RGDyK) for integrin binding; TPP-modified *MoS_2_–Ti_3_C_2_* for mitochondrial localization; cancer cell membrane cloaks; bacterial colonization of hypoxia enabling cavitation.	Multilevel targeting integrates ligand affinity, biomimetic interfaces, and organelle-specific localization.	[34,38,56,69,76]
Integrated Synergies	*MoO_x_* (PCE 51.5%, O_2_ production, GSH depletion, *^1^O_2_/·OH* generation); *Fe*-doped sulfides (NIR-II PTT + Fenton + ferroptosis); AIE agents combining emission, ROS, PTT, and mitochondrial targeting.	Multifunctional constructs support self-reinforcing mechanistic cascades that amplify therapeutic outcome.	[33,40,67]

**Table 3:** Overview of Diagnostic and Imaging Modalities in Nano-theranostic Platforms.

Diagnostic & Imaging Capabilities	Representative Examples	Expert Insights	Representative Refs.
Fluorescence Imaging (FL, NIR-II, Two-Photon, Lifetime)	NIR-II vascular mapping and bone-targeted diagnostics; apoptosis tracking via two-photon and lifetime FL; activatable probes responsive to pH, GSH, *H_2_O_2_*, or hypoxia; RNA-targeted nano sensors enabling mRNA/miRNA discrimination.	NIR-II imaging enhances penetration depth and tumor–background contrast; activatable probes provide stimulus-specific selectivity; RNA-level FL sensors add molecular-resolution precision to tumor characterization.	[2,12,14,16,24,34,37,39,50,78,79,81,82,83,84]
Photoacoustic/Optoacoustic Imaging (PA, PAM, MSOT)	NIR-II PAI and multispectral optoacoustic tomography for high-resolution tumor delineation; monitoring of apoptosis and vascular remodeling; TME-responsive probes activated by MMPs, NTR, or *H_2_S*.	PA combines optical absorption with ultrasound detection to achieve deep, high-contrast imaging; biochemical activation improves specificity for hypoxia, enzymatic activity, and redox gradients.	[13,15,31,35,41,54,77,82,85,86,87,88,89,90]
Magnetic Resonance Imaging (MRI: T_1_, T_2_, Dual, ^19^F)	*Mn-*, *Fe-*, and *Gd*-based T_1_/T_2_ contrast enhancers; dual-mode T_1_/T_2_ agents; activatable probes responsive to pH, GSH, and redox state; ^19^F MRI permitting background-free quantification.	Dual-mode probes minimize diagnostic ambiguity; ^19^F imaging offers high specificity without endogenous interference; elevated relaxivity values surpass those of standard clinical formulations.	[5,6,18,32,59,64,74,75,91,92,93,94]
Computed Tomography (CT)	High-Z nanomaterials (*Au*, *Pt*, *Bi*, *W*) for improved X-ray attenuation; CT combined with MRI and FL for structural–molecular integration.	CT provides precise anatomical mapping; high-Z nano agents frequently double as therapeutic photothermal or radio sensitizing components, enabling integrated diagnostics and therapy.	[17,29,42,56,73,87,95,96,97]
Ultrasound Imaging (US, HIFU Guidance)	PFH-loaded, aptamer-modified nanodroplets; bubble-generating constructs; engineered bacteria expressing gas vesicles for endogenous US contrast.	US gains functional depth when coupled with PA/MR or biologically engineered contrast sources; supports real-time therapeutic navigation, including HIFU ablation.	[4,22,30,38,63,99]
Multimodal Imaging Integration	Tri- and tetra-modal platforms combining MRI, CT, FL, PA, and US within a single construct.	Multimodal systems unify anatomical, molecular, and functional imaging, offering comprehensive intraoperative and longitudinal diagnostic guidance.	[29,42,48,73,98,100]
Functional & Molecular Diagnostics	Imaging of apoptosis, hypoxia, perfusion, ROS, GSH, H_2_O_2_, H_2_S; mRNA/miRNA-responsive nanoprobes for molecular fingerprinting.	Diagnostics evolve from passive contrast to active biosensing, enabling real-time mapping of metabolic stress, microenvironmental gradients, and gene-level markers.	[3,12,14,25,39,41,60,77,84,90,94,101]

**Table 4:** Refined Version—Therapeutic Modalities and Representative Nanoplatforms.

Therapeutic Capability	Representative Examples	Expert Insights	Representative Refs.
PDT + PTT Synergy	*S*, *N*-doped CDs; *HA-TiO_2_@MWCNTs/HMME*; iodinated aza-BODIPY nanoparticles	PDT/PTT combinations strengthen localized cytotoxicity, while catalytic components help sustain PDT efficacy under hypoxia.	[6,14,56,68,76]
Chemo–Photo Combinations	*DOX-Pt@Au@ZIF-8*; MMP-responsive Au NPs; polymeric micelles	Co-delivery and stimulus-triggered release enable spatially regulated chemo–photo activation and reduced systemic toxicity.	[17,30,42,89,102]
CDT and Ferroptosis	PBAM MOFs; *Fe-doped MoO_x_*; *Cu/CC* nanocomposites; IrO_X_-P cascades	Redox-catalytic ROS amplification enhances phototherapy, while iron-dependent ferroptosis provides irreversible lipid damage.	[1,13,18,83,88]
Immunotherapy + Local Modalities	*ICG-CpG@MOF*; *FYH-PDA-DOX*; biomimetic metallacages	Local ICD induction primes systemic antitumor immunity and complements immune checkpoint blockade strategies.	[34,53,69,100,104]
Radiotherapy & Acoustic Synergies	PEGylated *W-TiO_2_; BiVO_4_/Fe_3_O_4_@PDA*; *F3-PLGA@MB/Gd*	Thermo-radiotherapy improves RT efficacy in hypoxic tissues, while acoustic platforms integrate SDT, PTT, and mechanical drug release.	[23,29,38,87,105]
Metabolic & Gas Therapies	GOx-based nano factories; P1-CapNO; MnCO@CuS	Metabolic disruption and gas signaling (NO, CO, H_2_) modulate the TME and synergize with oxidative and photothermal therapies.	[12,36,75,106,107]
Theranostic Integration	DCDM nanoparticles; DUPM constructs; persistent luminescence probes	Embedded imaging functions provide real-time feedback, enabling adaptive, stimulus-responsive therapeutic control.	[3,40,58,73,98,108]

**Table 5:** In Vivo Outcomes & Translational Significance.

In Vivo Outcomes & Translational Significance	Examples/Explanations	Expert Insights	Representative Refs.
Complete tumor ablation & recurrence prevention	NM-NPs achieved total tumor elimination; DAA nanoparticles prevented relapse; *DOX/PPy-ELP-F3* ablated tumors without systemic toxicity.	Complete tumor clearance is attainable through optimized nanoplatform engineering—an essential benchmark for durable, relapse-free cancer therapy.	[9,78,109]
Metastasis suppression & systemic control	mPEG-PAAV micelles eradicated primary tumors and suppressed lung metastases; ABI Nys inhibited bone destruction and metastasis; *m@MTT* suppressed both local and distant lesions.	Demonstrating efficacy against metastatic progression strengthens translational relevance, addressing the most fatal dimension of cancer.	[31,69,82]
Multimodal & synergistic therapies	*DOX-Pt-tipped Au@ZIF-8* enabled potent chemo-phototherapy; *Fe^3+^-DOX* nanoliposomes triggered apoptosis + ferroptosis; *Cu/CC* assemblies enabled trimodal ROS-based therapy.	Synergy-driven designs overcome resistance mechanisms and achieve deeper, more durable tumor regression than monotherapies.	[1,17,83]
Immune activation & checkpoint synergy	FYH-PDA-DOX induced robust T-cell responses; *TAPP-GCP@TCPP@BSA* promoted ICD and synergized with PD-L1 blockade; Albumin-based nanocomposites regressed primary and metastatic TNBC lesions.	Nanoplatforms can function as powerful immune sensitizers, amplify checkpoint blockade efficacy and enable systemic tumor control.	[104,111,112]
TME responsiveness & resistance avoidance	*H_2_S*-activatable MOFs selectively ablated orthotopic colon tumors; P1-CapNO overcame hypoxia-induced PDT resistance; *AUC-GOx/Cel* nano factories enhanced CDT via catalytic feedback.	Leveraging TME-driven activation enhances precision, reduces off-target toxicity, and helps overcome adaptive resistance in heterogeneous tumors.	[12,36,90]
Imaging-guided precision & theranostic integration	*UCNP@SiO_2_-MB@PDA* enabled NIR-guided PDT/PTT; *PC61BA-*(*Gd-DO3A*)*/HSA* improved MRI-guided surgical precision; *MnCO@CuS* provided MRI-guided gas/photo-chemo dynamic therapy.	Integrating diagnostics with therapy transforms imaging into a real-time decision tool, enhancing spatial accuracy and treatment adaptability.	[16,59,75]
Safety, biocompatibility & clearance	PEGylated WTO nanoparticles were completely cleared within 30 days; IABN nanoparticles demonstrated low systemic toxicity; DUPM nanomedicine achieved 94.43% inhibition with strong biocompatibility.	Favorable biodistribution, biodegradability, and low toxicity establish a strong foundation for clinical translation.	[40,68,87]

**Table 6:** Translational Decision Framework for Nanotheranostics Platforms.

Therapeutic/Platform Theme	Rationale (Clinical Need)	Mechanistic Core (How It Works)	Current Evidence (Preclinical)	Candidate Populations/Contexts	Outcome Signals (What We See)	Practice-Directed Next Steps	Future Gaps & Priorities	Representative Refs.
PDT + PTT Synergy Hub	Overcome chemo/radio-resistance; precise local control with minimal systemic toxicity	Light-driven ROS (PDT) + hyperthermia (PTT); catalytic O_2_ supply via catalase, MOx, or PDA-hybrid shells to offset hypoxia	Complete tumor ablation in murine models; sustained ROS generation in MPDA/MON/PDA-ICG constructs	TNBC, hepatic tumors, hypoxic & heterogeneous solid masses	Full preclinical eradication with low toxicity	Standardize NIR/NIR-II parameters; integrate intraoperative imaging guidance	Depth constraints; inorganic residue long-term safety; thermal gradients	[6,14,56,68,76]
Chemo–Photo Platforms (PTT/PDT + Drug)	Minimize systemic chemotherapy burden via on-demand localized release	Heat/pH/MMP-responsive release; co-localized chemo + photoablation	Single-laser chemo–photo synergy; ligand-directed artemisinin delivery; robust micelle & mesoporous control	Large solid tumors; lesions near sensitive anatomy	Enhanced inhibi-tion vs. mono-therapy; strong cooperative effects	Optimize release–light timing; image-guided dose planning	Scale-up issues; EPR variability; risk of premature activation	[17,30,31,42,89]
CDT & Ferroptosis Catalytic Amplifiers	Address hypoxia and redox-rich, drug-resistant tumors	Fenton/Fenton-like ·OH generation; GSH depletion; lipid peroxidation (ferroptosis); NIR-II enhanced catalysis	MRI-guided CDT; trimodal ROS cascades; ferroptosis + apoptosis/ICD	Drug-resistant, hypoxic tumors with high antioxidant buffering	Potentiated tumor kill; catalytic self-reinforcement	Develop ferroptosis biomarkers; pair with low-dose PDT/PTT	Off-target oxidative injury; metal fate & persistence; variable redox microenvironments	[1,13,18,33,40,74,83,88]
Immunotherapy + Local Ablation	Translate local ablation into systemic immune control	ICD induction; checkpoint synergy (PD-L1); CpG or adjuvant-reinforced immune activation; macrophage repolarization	Distant tumor suppression; cold-tumor reprogramming; improved checkpoint outcomes	Metastatic TNBC; immunologically “cold” phenotypes	Systemic T-cell activation; abscopal-like responses	Synchronize ICD with checkpoint dosing; integrate immune monitoring	Durability across heterogeneous immune states; autoimmune risks	[34,53,69,100,104,111,112]
RT & Acoustic Synergies (SDT, HIFU, FUAS)	Treat deep, poorly perfused lesions; combine mechanical & thermal modalities	SDT ROS formation; thermo-radiotherapy; HIFU-triggered drug release; biological cavitation via engineered microbes	NIR-II PTT + RT synergy; SDT/HIFU platforms with activatable delivery	Deep-seated tumors; bone/brain-adjacent malignancies	Enhanced ablation; multi-modal image guidance	Co-register RT/US with MRI/PAI; define thermal–dose thresholds	Workflow complexity; cavitation unpredictability; tissue-specific acoustics	[23,29,38,87,105]
Metabolic & Gas Therapies (Starvation/NO/CO/H_2_)	Reprogram TME metabolism and Vaso dynamics to overcome resistance	GOx-mediated glucose depletion + H_2_O_2_ amplification; NO/CO/H_2_ release regulating perfusion, stress pathways, or inflammation	Dual H_2_O_2_-amplifying nano factories; NO-augmented PTT; CO-integrated PTT/CDT	Hypoxic, glycolytic tumors; metastatic niches	Strong synergy with PDT/PTT/CDT; reduced inflammatory damage	TME-mapping for patient stratification; safety thresholds for gas donors	Tumor metabolic plasticity; gas diffusion control; vascular off-target effects	[12,36,75,106,107]
Theranostics & Imaging-Integrated Platforms	Need real-time monitoring, adaptive dosing, surgical margin clarity	MRI/CT/PA/FL/NIR-II integrated with ROS/O_2_/GSH-responsive activation; persistent luminescence for excitation-free tracking	NIR-II/CT/MRI-guided PDT/PTT/CDT; dynamic adaptive imaging	Candidates requiring surgical navigation or ambiguous margins	Higher spatial precision; multimodal real-time feedback	Define imaging–response endpoints; develop closed-loop control algorithms	Scanner variability; relaxivity–safety trade-offs; standardization barriers	[3,16,40,58,59,73,75,94,98,108]
Materials & Manufacturability (Cross-Cutting Theme)	Scale, safety, and regulatory feasibility	Intrinsic multifunctionality via doping/defects; PDA/PLGA; green synthesis; reduced component complexity	High PCE and catalytic efficiency with simplified constructs; 30-day clearance in some inorganic systems	Outpatient & OR-compatible systems; repeat-dose settings	Strong biocompatibility; low dark toxicity	Prioritize single-molecule or few-component platforms; GMP-ready synthesis	Chronic biodistribution; patient variability in EPR; regulatory complexity	[20,23,29,30,40,67,68,69,87]

## Data Availability

No new data were created or analyzed in this study.

## Abbreviations

PTT: photothermal therapy; PDT: photodynamic therapy; CDT: chemo dynamic therapy; ICD: immunogenic cell death; RT: radiotherapy; SDT: sonodynamic therapy; HIFU/FUAS: high-intensity/focused ultrasound ablation; TME: tumor microenvironment; EPR: enhanced permeability and retention; NIR-II: second near-infrared window.
